# A longitudinal study of the associations of children's body mass index and physical activity with blood pressure

**DOI:** 10.1371/journal.pone.0188618

**Published:** 2017-12-19

**Authors:** Corrie Macdonald-Wallis, Emma Solomon-Moore, Simon J. Sebire, Janice L. Thompson, Deborah A. Lawlor, Russell Jago

**Affiliations:** 1 Centre for Exercise, Nutrition & Health Sciences, School for Policy Studies, University of Bristol, Bristol, United Kingdom; 2 School of Sport, Exercise and Rehabilitation Sciences, University of Birmingham, Birmingham, United Kingdom; 3 MRC Integrative Epidemiology Unit at the University of Bristol, Bristol, United Kingdom; 4 School of Social and Community Medicine, University of Bristol, Bristol, United Kingdom; Shanghai Institute of Hypertension, CHINA

## Abstract

Childhood blood pressure is a marker of cardiovascular disease risk in later life. We examined how body mass index (BMI) and physical activity, and changes in these, are associated with blood pressure in primary school-aged children. Data are from 1223 children aged 9 years (Year 4) in Bristol, UK, 685 of whom had been assessed at 6 years (Year 1). Child height and weight were measured, and children wore accelerometers for five days, from which average counts per minute, and moderate-to-vigorous-intensity physical activity and sedentary minutes per day were derived. At age 9 years, blood pressure was measured. Multiple imputation of missing data and adjusted linear regression models were used to examine associations. Child BMI at 9 years was cross-sectionally associated with higher systolic (SBP) and diastolic (DBP) blood pressure (mean difference [95% CI]: 1.10 [0.34, 1.87] mmHg and 0.86 [0.13, 1.60] mmHg, respectively, per SD of BMI). Prospective associations of BMI at age 6 with blood pressure at age 9 were consistent with these cross-sectional associations. However, change in BMI between 6 and 9 years was not strongly associated with subsequent SBP or DBP (0.68 [-0.61, 1.98] mmHg and 1.23 [-0.09, 2.54] mmHg, respectively). There was little evidence that physical activity or sedentary time were associated with blood pressure in either cross-sectional or prospective analyses. Greater childhood BMI is associated with higher blood pressure, and this association persists over several years. Prevention of excessive bodyweight from early childhood may be important in stemming the development of cardiovascular risk.

## Introduction

Blood pressure in childhood has been shown to track into adulthood [1], and is a marker of cardiovascular disease risk in later life [2, 3]. In adults, body mass index (BMI) and physical inactivity are key modifiable risk factors for hypertension, and interventions aimed at increasing time spent being physically active have shown effectiveness in reducing both BMI and blood pressure [4, 5].

In children, cross-sectional studies have generally shown a strong positive association between BMI and blood pressure [6–11], but few studies have examined prospective associations of BMI during childhood with later blood pressure [12–14]. Those that have suggest that children who transition from normal to overweight [12, 13], or have a steeper than average trajectory of BMI change [12, 14], have a greater risk of high blood pressure later in childhood or adolescence. Longitudinal studies have, however, mainly focused on blood pressure measured in the second decade of life, and little is known about how adiposity relates prospectively to blood pressure in primary (elementary) school-aged children, and whether overweight and obesity have long-lasting or transient effects on blood pressure at this age.

Studies that have examined the association between physical activity and blood pressure in children have had inconsistent findings, with some showing that more time spent being active [15–25], and less time spent in sedentary behaviours [16, 17, 25, 26], are associated with lower blood pressure or reduced risk of hypertension, and others finding no association between physical activity and blood pressure [27–33]. Several of these studies have been limited by self-reported measures of physical activity and sedentary time, both of which are subject to recall bias [15, 18, 19, 32]. Two studies that examined the prospective association of accelerometer-assessed physical activity in childhood showed little association with subsequent blood pressure [17, 30].

We investigated the cross-sectional and longitudinal associations between BMI and accelerometer-assessed physical activity, and change in these, with blood pressure in primary (elementary) school-aged children. We further aimed to assess whether any observed prospective associations of physical activity with blood pressure were mediated by the child’s BMI.

## Methods

B-Proact1v is a longitudinal study examining changes in children’s physical activity and sedentary behaviours as they progress through primary school [34, 35]. In 2012–2013, 1299 Year 1 children (median age: 6 years) were recruited from 57 schools in greater Bristol, UK (total number of eligible children: 2600; recruitment rate: 50.0%). Following this, data were collected from 1223 Year 4 children (median age: 9 years) from 47 of the original schools between March 2015 and July 2016 (total number of eligible children: 2047; recruitment rate: 59.7%) [35]. This included 685 children from the original sample. The study received ethical approval from the School for Policy Studies Ethics Committee at the University of Bristol, and written parental consent was received for all participants [36].

### Measurement of BMI and blood pressure

Children’s height and weight were measured at schools at age 6 and 9 years, with blood pressure also measured at 9 years. Height was measured to the nearest 0.1cm using a SECA Leicester stadiometer, and weight was measured to the nearest 0.1kg using a SECA 899 digital scale (HAB International, Northampton). BMI was derived as weight (kg)/ height (m)^2^ and converted to an age- and gender-specific standard deviation score based on UK 1990 growth centiles [37]. This was then categorised as normal weight (<85th percentile) or overweight/obese (≥85th percentile). Blood pressure was measured using an Omron 907 Professional Blood Pressure Monitor [38] with a small or medium cuff (OMRON Corporation, Kyoto, Japan), three times, one-minute apart, in the left arm with the child seated and after a three-minute rest period. The average of all three measurements was used in analysis, or the average of two measurements for the 7% of children who did not have a third measure. Systolic and diastolic hypertension were defined as the systolic or diastolic blood pressure, respectively, that was ≥95^th^ percentile using US children’s blood pressure references that have been standardized for age, gender, and height [39]. US reference charts were used because of the lack of such population references for UK children.

### Accelerometer measures of physical activity

Children wore a waist-worn ActiGraph wGT3X-BT accelerometer for five days, including two weekend days, at both timepoints. Accelerometer data were processed using Kinesoft (v3.3.75; Kinesoft, Saskatchewan, Canada). At least three valid days of data were required for inclusion in analysis, where a valid day was defined as ≥500 minutes of data, after excluding intervals of ≥60 minutes of zero counts allowing up to two minutes of interruptions. We obtained the average number of counts per minute (CPM) which provides an indication of the volume of physical activity in which the children engaged over all valid days. We used the Evenson et al., population-specific cut points for children [40], to derive the average number of moderate-to-vigorous-intensity physical activity (MVPA) and sedentary minutes per day.

### Parent and child characteristics

One or both of the children’s parents were recruited to the study. Both parents (if participating) completed questionnaires including information about their own date of birth, height, and weight, from which maternal and paternal age and BMI were derived. To maximise available data, if a parent participated in one study year but not the other, their reported BMI in one year was used throughout. The first parent reported child gender and date of birth. Where the child’s date of birth was missing (21% of all children), the median age was assigned (6.0 years at Year 1, 9.0 years at Year 4). In Year 4, the first parent questionnaire also asked if either of the child’s biological parents had ever been informed that they had high blood pressure. Indices of Multiple Deprivation (IMD) scores, based upon the English Indices of Deprivation,[41] were assigned to each family based on their reported home postcode.

### Statistical analysis

Linear regression models were used to examine the associations between the child’s BMI z-score, BMI category, CPM, MVPA and sedentary time, and change in these, with blood pressure. Model 1 was unadjusted for BMI z-score or BMI category, and adjusted for child age, gender and height for activity measures. For the models with change in BMI or activity measures, we adjusted for baseline (age 6 years) measures of the risk factor being examined. In Model 2, we additionally adjusted for IMD, maternal and paternal BMI, and parental high blood pressure. For potential mediation in the cross-sectional and prospective analyses of activity measures by the child’s BMI we additionally adjusted for the child’s BMI z-score at age 9 years in Model 3. A similar series of models were used to explore cross-sectional and prospective associations with childhood hypertension. We undertook all analyses with girls and boys combined; and separately by gender. Where there was evidence of gender differences (notable differences in point estimates and/or statistical evidence for interaction) we present results stratified by gender; otherwise only combined results are presented. To account for clustering of children within schools and the associated potential to underestimate the standard errors which are used to compute the 95% confidence intervals and p-values, robust standard errors, which took account of the school-level clustering, were used for all models.

#### Dealing with missing data

Among both the 1223 children used in cross-sectional analyses and the 685 used in prospective analyses there were small amounts of missing data for risk factors, blood pressure and/or confounders (Table 1). This varied from 0 (e.g., for child age and gender) to 16% (for CPM, MVPA, and sedentary time) in the cross-sectional analyses and 29% (for change in CPM, MVPA, and sedentary time) in the prospective analyses. We used multiple imputation for these missing data to increase power and minimise selection bias in our findings. This was done separately for cross-sectional and prospective analyses. Thus, for cross-sectional analyses between BMI or physical activity at age 9 and blood pressure (at age 9) we imputed to the 1223 children who participated at age 9, and for prospective analyses examining associations between BMI or physical activity at age 6, or change in these between 6 and 9 years, and blood pressure we imputed to the 685 children who took part at both time points.

**Table 1:** Characteristics of children who participated in the study only at age 9 years and those who participated at both ages 6 and 9 years in the observed and multiple imputation datasets.

Child Characteristic				Children who participated at age 9 (Total N = 1223)*			Children who participated at ages 6 and 9 (Total N = 685)^†^		
				Observed data		Imputed (N = 1223)	Observed data		Imputed (N = 685)
				N available	Mean (SD) or %	Mean (SD) or %	N available	Mean (SD) or n (%)	Mean (SD) or %
Systolic blood pressure at 9 years (mmHg)				1201	106.2 (11.9)	106.3 (12.0)	670	105.7 (11.4)	105.7 (11.5)
Diastolic blood pressure at 9 years (mmHg)				1201	70.7 (11.1)	70.7 (11.2)	670	70.4 (10.5)	70.4 (10.6)
Systolic hypertension		No		1034	86.1	86.0	581	86.7	86.6
		Yes		167	13.9	14.0	89	13.3	13.4
Diastolic hypertension		No		960	79.9	79.8	541	80.7	80.8
		Yes		241	20.1	20.2	129	19.3	19.2
Counts per minute at 6 years							560	713.9 (171.4)	712.6 (174.1)
Counts per minute at 9 years				1026	612.6 (197.9)	611.4 (200.3)	584	619.4 (203.0)	619.0 (208.0)
Change in counts per minute between 6 to 9 years							488	-87.6 (212.6)	-93.6 (217.9)
MVPA at 6 years (mins/day)							560	67.6 (19.7)	67.4 (20.0)
MVPA at 9 years (mins/day)				1026	61.6 (21.9)	61.5 (22.1)	584	62.0 (21.6)	62.1 (22.1)
Change in MVPA between 6 to 9 years (mins/day)							488	-5.0 (21.5)	-5.3 (22.2)
Sedentary time at 6 years (mins/day)							560	361.7 (58.5)	361.2 (59.7)
Sedentary time at 9 years (mins/day)				1026	445.4 (115.4)	445.2 (118.1)	584	444.4 (114.3)	445.6 (119.2)
Change in sedentary time between 6 to 9 years (mins/day)							488	79.7 (110.5)	84.4 (126.6)
BMI z-score at 6 years							670	0.20 (0.93)	0.20 (0.94)
BMI category at 6 years		Normal					558	83.3	83.0
		Overweight					112	16.7	17.0
BMI z-score at 9 years				1217	0.35 (1.07)	0.34 (1.07)	682	0.33 (1.06)	0.33 (1.06)
BMI category at 9 years		Normal		913	75.0	75.0	514	75.4	75.3
		Overweight		304	25.0	25.0	168	24.6	24.7
Change in BMI z-score between 6 to 9 years							667	0.13 (0.67)	0.13 (0.68)
Change in BMI category between 6 to 9 years		Normal-Normal					481	72.1	71.7
		Normal-Overweight					75	11.2	11.3
		Overweight -Normal					23	3.4	3.6
		Overweight -Overweight					88	13.2	13.4
Gender		Boy		556	45.5	45.5	323	47.2	47.2
		Girl		667	54.5	54.5	362	52.8	52.8
Age in Year 1 (years)							685	6.0 (0.4)	6.0 (0.4)
Age in Year 4 (years)				1223	9.0 (0.4)	9.0 (0.4)	685	9.0 (0.4)	9.0 (0.4)
Height at 6 years (m)							670	1.16 (0.06)	1.16 (0.06)
Height at 9 years (m)				1217	1.34 (0.07)	1.34 (0.07)	682	1.34 (0.06)	1.34 (0.06)
IMD score at 6 years							627	14.4 (12.6)	14.4 (12.7)
IMD score at 9 years				1204	15.9 (14.1)	15.9 (14.1)	673	15.3 (14.0)	15.3 (14.0)
Mother’s age at age 6 (years)							516	37.6 (5.3)	37.5 (5.6)
Mother’s age at age 9 (years)				790	40.6 (5.4)	40.4 (5.7)			
Father’s age at age 6 (years)							344	40.3 (6.0)	39.8 (7.4)
Father’s age at age 9 (years)				487	43.2 (5.9)	43.0 (7.0)			
Mother’s BMI at 6 years (kg/m^2^)							575	25.1 (4.5)	25.0 (4.7)
Mother’s BMI at 9 years (kg/m^2^)				875	25.6 (5.1)	25.7 (5.4)			
Father’s BMI at 6 years (kg/m^2^)							399	26.3 (3.9)	26.4 (4.7)
Father’s BMI at 9 years (kg/m^2^)				588	26.3 (3.8)	26.5 (4.5)			
Parent had high blood pressure		No		827	83.7	81.8	473	83.3	81.2
		Yes		161	16.3	18.2	95	16.7	18.8

* Used for analysis of physical activity and BMI at 9 years with blood pressure. Characteristics not included in this analysis are shaded out in this column.

^†^ Used for analysis of physical activity and BMI at 6 years, as well as change in physical activity and BMI between 6 and 9 years, with blood pressure.

In both imputation models, we included child blood pressure at age 9, child BMI z-score, CPM, MVPA, sedentary time, age and height at age 6 and 9, child gender, IMD, maternal and paternal age and BMI, parental high blood pressure, and the child’s school. Changes in child BMI z-score and physical activity between 6 and 9 years, the child’s BMI category, and systolic and diastolic hypertension were imputed passively. This meant that all outcomes, exposures, potential confounders, and additional variables that may be predictive of missingness or of the missing values themselves were included in imputation models. We created 20 imputed datasets using 20 cycles of regression switching and combined regression coefficients across imputed datasets using Rubin’s rules [42].

Regression analyses were repeated restricting to children who had complete data on all exposures, outcomes and covariables for comparison with the multiple imputation analysis (N = 370 and 275, in cross-sectional and prospective analyses respectively. All analyses were performed in Stata version 14.0 (StataCorp, 2015).

## Results

The characteristics of all children and parents who participated at age 9, and the subset who additionally took part at age 6, in the observed and multiple imputation datasets are shown in Table 1. The subset that took part in both years were comparable to the whole age 9 sample, and in both years the distributions of all characteristics were very similar in multiple imputation and observed data. The mean systolic blood pressure (SBP) for all 9-year-old children was 106 mmHg (14% had systolic hypertension) and mean diastolic blood pressure (DBP) was 71 mmHg (20% had diastolic hypertension). The frequency distributions for BMI z-score at 6 years and 9 years overlaid and for MVPA minutes/day at 6 years and 9 years overlaid are shown in S1 and S2 Figs respectively.

### BMI and blood pressure

For some associations involving BMI exposure variables and blood pressure there was evidence of an interaction between child gender and BMI, so regression coefficients are presented for boys and girls separately, as well as for both genders combined.

The cross-sectional and prospective associations of BMI with blood pressure outcomes are shown in Table 2. In cross-sectional analyses, higher BMI was associated with higher SBP and DBP in both the unadjusted and confounder-adjusted model and being overweight/obese compared with normal weight was also associated with higher SBP and DBP in both models. These associations were similar in boys and girls. Prospective analyses (examining associations of BMI at age 6 years with SBP and DBP at age 9 years) were broadly consistent with these cross-sectional results, with the exception of associations between overweight/obese versus normal weight for which there appeared to be gender differences. Mean SBP and DBP were higher at 9 years for girls who were overweight compared with normal weight at 6 years, but there was little difference for boys.

**Table 2:** Cross-sectional and prospective associations of BMI with blood pressure at age 9 years in the multiple imputation data.

Exposure		Systolic blood pressure (mmHg) at 9 years			Diastolic blood pressure (mmHg) at 9 years		
		Mean difference	Mean difference	Mean difference	Mean difference	Mean difference	Mean difference
		(95% CI)	(95% CI)	(95% CI)	(95% CI)	(95% CI)	(95% CI)
**BMI z-score at 9 years**		All (N = 1223)	Boys (N = 556)	Girls (N = 667)	All (N = 1223)	Boys (N = 556)	Girls (N = 667)
(per SD of BMI)^*^							
	Model 1	1.11 (0.41, 1.81)	1.81 (0.97, 2.64)	0.63 (-0.41, 1.66)	0.89 (0.20, 1.59)	1.34 (0.58, 2.11)	0.52 (-0.51, 1.55)
	Model 2	1.10 (0.34, 1.87)	2.04 (1.09, 2.99)	0.41 (-0.72, 1.55)	0.86 (0.13, 1.60)	1.66 (0.76, 2.56)	0.28 (-0.81, 1.37)
P value for gender interaction		0.06			0.19		
Overweight (vs normal weight) at 9 years^*^							
	Model 1	2.05 (0.61, 3.49)	2.32 (0.24, 4.40)	2.15 (0.40, 3.91)	2.04 (0.55, 3.52)	1.90 (-0.24, 4.04)	2.11 (0.18, 4.04)
	Model 2	1.94 (0.47, 3.41)	2.46 (0.21, 4.71)	1.76 (-0.09, 3.62)	1.91 (0.36, 3.47)	2.23 (-0.01, 4.48)	1.66 (-0.38, 3.70)
P value for gender interaction		0.80			0.85		
**BMI z-score at 6 years**		All (N = 685)	Boys (N = 323)	Girls (N = 362)	All (N = 685)	Boys (N = 323)	Girls (N = 362)
**(per SD of BMI)**^†^							
	Model 1	1.26 (0.19, 2.34)	1.61 (0.14, 3.08)	1.00 (-0.27, 2.28)	0.98 (0.05, 1.90)	1.22 (-0.10, 2.54)	0.72 (-0.39, 1.84)
	Model 2	1.43 (0.31, 2.54)	2.00 (0.53, 3.48)	0.92 (-0.48, 2.32)	1.07 (0.12, 2.03)	1.52 (0.14, 2.89)	0.67 (-0.55, 1.88)
P value for gender interaction		0.47			0.58		
**Overweight (vs normal weight) at 6 years**^†^							
	Model 1	1.67 (-1.21, 4.55)	-0.31 (-4.29, 3.66)	3.40 (0.21, 6.59)	1.71 (-0.96, 4.37)	-0.88 (-4.90, 3.13)	3.79 (0.95, 6.64)
	Model 2	1.99 (-0.89, 4.86)	0.53 (-3.37, 4.42)	3.32 (0.09, 6.55)	1.87 (-0.89, 4.63)	-0.32 (-4.69, 4.04)	3.78 (0.73, 6.82)
P value for gender interaction		0.10			0.03		

* Model 1 is unadjusted; Model 2 is adjusted for the household IMD score, maternal BMI, paternal BMI at 9 years and parental high blood pressure

^†^ Model 1 is unadjusted; Model 2 is adjusted for the household IMD score, maternal BMI, paternal BMI at 6 years and parental high blood pressure

There were positive cross-sectional and prospective associations of BMI and overweight/obese with systolic and diastolic hypertension, with associations being somewhat stronger for diastolic hypertension (Table 3). There was no strong evidence that these associations differed between boys and girls.

**Table 3:** Cross-sectional and prospective associations of BMI with hypertension at age 9 years in the multiple imputation data.

Exposure		Systolic hypertension at 9 years			Diastolic hypertension at 9 years		
		Odds ratio	Odds ratio	Odds ratio	Odds ratio	Odds ratio	Odds ratio
		(95% CI)	(95% CI)	(95% CI)	(95% CI)	(95% CI)	(95% CI)
**BMI z-score at 9 years**		All (N = 1223)	Boys (N = 556)	Girls (N = 667)	All (N = 1223)	Boys (N = 556)	Girls (N = 667)
(per SD of BMI)*							
	Model 1	1.16 (1.01, 1.34)	1.31 (1.07, 1.60)	1.05 (0.85, 1.30)	1.22 (1.05, 1.42)	1.32 (1.11, 1.55)	1.14 (0.92, 1.42)
	Model 2	1.15 (0.98, 1.35)	1.36 (1.07, 1.72)	0.98 (0.78, 1.25)	1.23 (1.04, 1.46)	1.43 (1.16, 1.76)	1.12 (0.88, 1.43)
P value for gender interaction		0.15			0.33		
Overweight (vs normal weight) at 9 years*							
	Model 1	1.41 (1.03, 1.93)	1.52 (0.92, 2.50)	1.37 (0.90, 2.09)	1.65 (1.20, 2.26)	1.63 (1.01, 2.63)	1.63 (1.10, 2.43)
	Model 2	1.37 (0.98, 1.90)	1.55 (0.91, 2.64)	1.22 (0.78, 1.92)	1.68 (1.19, 2.37)	1.80 (1.08, 3.01)	1.59 (1.02, 2.47)
P value for gender interaction		0.74			0.93		
**BMI z-score at 6 years**		All (N = 685)	Boys (N = 323)	Girls (N = 362)	All (N = 685)	Boys (N = 323)	Girls (N = 362)
**(per SD of BMI)**^†^							
	Model 1	1.13 (0.91, 1.42)	1.27 (0.93, 1.74)	1.02 (0.76, 1.36)	1.31 (1.08, 1.60)	1.44 (1.10, 1.88)	1.22 (0.95, 1.56)
	Model 2	1.19 (0.94, 1.50)	1.42 (1.03, 1.96)	0.97 (0.69, 1.35)	1.35 (1.10, 1.65)	1.61 (1.18, 2.19)	1.20 (0.93, 1.57)
P value for gender interaction		0.30			0.39		
**Overweight (vs normal weight) at 6 years**^†^							
	Model 1	1.45 (0.86, 2.44)	0.97 (0.42, 2.24)	1.96 (1.11, 3.48)	1.59 (1.03, 2.45)	0.92 (0.39, 2.14)	2.17 (1.23, 3.83)
	Model 2	1.61 (0.93, 2.76)	1.19 (0.52, 2.76)	1.90 (1.02, 3.55)	1.65 (1.03, 2.62)	1.08 (0.43, 2.70)	2.15 (1.14, 4.04)
P value for gender interaction		0.14			0.10		

* Model 1 is unadjusted; Model 2 is adjusted for the household IMD score, maternal BMI, paternal BMI at 9 years and parental high blood pressure

^†^ Model 1 is unadjusted; Model 2 is adjusted for the household IMD score, maternal BMI, paternal BMI at 6 years and parental high blood pressure

**Fig 1:**
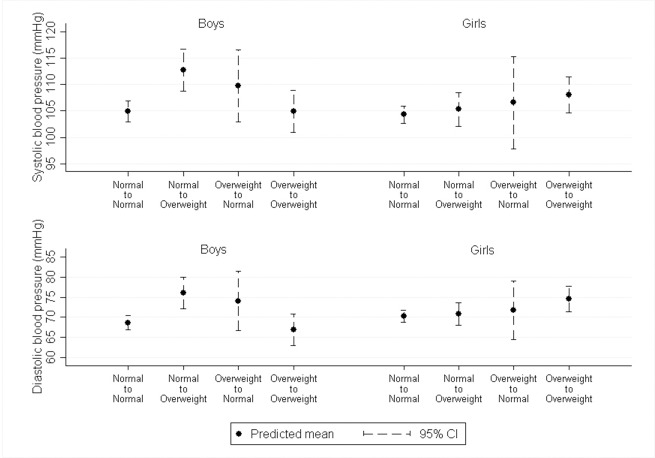
Predicted systolic and diastolic blood pressure by change in BMI category from age 6 to 9 years in multiple imputation data (N = 685)*. *Predictions are from regression models adjusted for household IMD score, maternal and paternal BMI and parental hypertension. The predicted values are for a child from a household with an IMD score of 15, maternal BMI of 25, paternal BMI of 26 (the mean values) and whose parents did not have hypertension. Boys: Normal to Normal (N = 232); Normal to Overweight (N = 31); Overweight to Normal (N = 22); Overweight to Overweight (N = 39); Girls: Normal to Normal (N = 247); Normal to Overweight (N = 42); Overweight to Normal (N = 14); Overweight to Overweight (N = 59).

Change in BMI was not strongly associated with SBP or DBP in any of the models (Fig 1 & S1 Table). However, children who increased their BMI category from normal to overweight/obese between 6 and 9 years had higher SBP and DBP at age 9 years, and these associations appeared to be stronger for boys. Additionally, girls who were overweight at both 6 and 9 years had higher SBP and DBP than girls who were normal weight at both years, but this difference was weaker in boys.

### Physical activity and blood pressure

Since there was no strong evidence of interactions between child gender and any of the physical activity measures in their associations with blood pressure (all p-values ≥ 0.05 and directions and magnitudes very similar in boys and girls for all analyses), we present results for both genders combined.

In cross-sectional analyses, there was no strong evidence of associations between CPM or MVPA and SBP or DBP in any of the models, and no evidence that sedentary time was associated with SBP (Table 4). There was a weak positive cross-sectional association of sedentary time with DBP (difference in mean per 10 minute greater time per day spent sedentary in the confounder-adjusted model: 0.05 mmHg (0.00, 0.11)), which did not appear to be mediated by BMI.

**Table 4:** Cross-sectional associations of physical activity with blood pressure at age 9 years in the multiple imputation data (N = 1223)*.

Exposure		Systolic blood pressure (mmHg) at 9 years			Diastolic blood pressure (mmHg) at 9 years		
		Mean difference	95% confidence interval	P-value	Mean difference	95% confidence interval	P-value
**Counts per minute at 9 years (per 100 cpm)**							
	Model 1	0.11	(-0.32, 0.53)	0.62	-0.08	(-0.47, 0.30)	0.66
	Model 2	0.09	(-0.34, 0.53)	0.66	-0.09	(-0.48, 0.29)	0.62
	Model 3	0.11	(-0.33, 0.55)	0.61	-0.08	(-0.47, 0.31)	0.68
**MVPA at 9 years (per 10 mins/day)**							
	Model 1	0.11	(-0.28, 0.49)	0.58	-0.04	(-0.40, 0.31)	0.81
	Model 2	0.11	(-0.27, 0.50)	0.56	-0.04	(-0.39, 0.31)	0.80
	Model 3	0.15	(-0.24, 0.54)	0.45	-0.01	(-0.36, 0.34)	0.94
**Sedentary time at 9 years (per 10 mins/day)**							
	Model 1	-0.01	(-0.06, 0.05)	0.81	0.05	(-0.01, 0.11)	0.08
	Model 2	0.00	(-0.06, 0.06)	0.93	0.05	(0.00, 0.11)	0.07
	Model 3	0.00	(-0.06, 0.06)	0.93	0.05	(-0.01, 0.11)	0.07

*Model 1 is adjusted for the child’s gender, age and height at age 9 years; Model 2 is additionally adjusted for household IMD score, maternal BMI, paternal BMI at 9 years and parental high blood pressure; Model 3 is additionally adjusted for mediation by the child’s BMI z-score at 9 years.

There was no strong evidence of prospective associations of CPM, MVPA or sedentary time measured, or that change in CPM, MVPA or sedentary time between 6 and 9 years with SBP or DBP (S3 Table).

### Analysis for those with complete data

Generally, the findings in the restricted complete case dataset were comparable to those in the multiple imputation data (S4–S9 Tables). However, some associations of accelerometer measurements with blood pressure appeared to emerge in this subset of the data: an inverse prospective association of CPM and positive association of sedentary time with DBP (S9 Table).

## Discussion

In this large cohort of primary-school children, we found similar cross-sectional and prospective associations of BMI with mean systolic and diastolic blood pressure and hypertension. These results suggest that higher BMI in early childhood (around 6 years) is associated with elevated blood pressure that persists for at least three years, and is robust to changes in both BMI and blood pressure that occur as children grow. In support of this, there was little evidence that change in BMI over the three-year period was associated with blood pressure at age 9 years. Transitioning from normal weight to overweight was associated with increased blood pressure only in boys. Conversely, there was little evidence that children’s physical activity or sedentary time at either age, or changes in these, were associated with blood pressure at age 9 years.

A meta-analysis including studies of children aged 5–15 years reported a difference of 4.5 mmHg for SBP and 2.6 mmHg for DBP for overweight compared with normal weight children [7]. These differences are slightly larger in magnitude than those we found in the current study (~1.9 mmHg difference between overweight and normal weight children for both SBP and DBP), potentially due to the broad age range covered by the meta-analysis, whereas we focused on a specific primary-school year-group. It is possible that differences in blood pressure associated with BMI become larger with age. In contrast to our lack of association between change in BMI and mean blood pressure, a large prospective study also from Bristol, UK (N = 5235; Avon Longitudinal Study of Parents and Children (ALSPAC)), found that change in BMI between age 9–12 years and 15–16 years was associated with greater SBP at 15–16 years, but no association with DBP [12]. Boys and girls in the ALSPAC cohort who transitioned from normal weight to overweight, or were overweight at both ages, were similarly associated with increased odds of high SBP [12]. In contrast, we found evidence for sex differences in these associations, with changing from normal weight to overweight being associated with the highest blood pressure in boys, but being overweight at both ages being associated with the highest blood pressure in girls. However, these sex differences often fail to replicate in other studies and may be due to chance, for instance a Dutch study (N = 1432) observed a different pattern of sex differences [13]. These differences may also be due to differences in age or birth cohort, the ALSPAC participants were born in the 1990s and thus have spent less of their lives in an obese environment than the current cohort who were born in 2006–2007.

Previous studies have also shown conflicting evidence regarding the association of physical activity with blood pressure at this age, with one Canadian study of high risk children (N = 536; at least one parent with obesity) finding inverse associations of CPM and MVPA and positive associations of sedentary time with DBP, but not SBP [16]. A, second, UK study of mixed-ethnicity 9-10-year-old children (N = 2049), also found some evidence of an inverse association of MVPA with DBP, but a weaker association with SBP [24]. Conversely, two Danish studies in 9–10 and 8-11-year-old children (N = 589 and 723 respectively), found no association of accelerometer-assessed MVPA and sedentary time with blood pressure [27, 30]. A longitudinal study of predominantly South Asian children (N = 427) found that greater average CPM at age 5–7 years was associated with lower blood pressure two years later, but MVPA was not [21]. These mixed findings suggest that physical activity may not be a key determinant of blood pressure in early childhood, with determinants such as BMI, height and genetic influences possibly being more important risk factors for higher blood pressure in the first decade of life.

One clinical implication from the findings in the current study is that measuring BMI in primary-school children is a suitable measure for identifying those at risk of future adverse cardiovascular risk profiles, and highlights the importance of prevention strategies aimed at reducing population levels of childhood adiposity. Although the differences in SBP and DBP between normal weight and overweight children in the current study may appear small (~1.9 mm Hg), in adults, a 2 mm Hg reduction in blood pressure is associated with a 6% reduction in coronary heart disease and a 15% reduction in stroke-related events [43].

### Strengths and limitations

Strengths of this study include the objective measurements of BMI and physical activity (via accelerometers) at two ages in childhood, three years apart, which allowed us to examine longitudinal associations of these exposures with subsequent blood pressure at age 9 years. The study is limited by a high proportion of missing parent data, since only one parent was required to participate in the study, and the parent taking part sometimes differed at the two time-points. We have used multiple imputation of missing data to increase precision and reduce selection bias in our coefficient estimates [44], and confidence intervals for coefficients in the imputed data were consistent with those found when restricting to children with complete data. As this is an observational study we cannot exclude residual confounding, for example by dietary factors such as salt, sugar or fat intake. The study is also limited by the absence of blood pressure data at age 6, meaning we were not able to examine change in blood pressure between 6 and 9 years. It is possible that the longitudinal associations observed between BMI and blood pressure were driven by those with higher BMI scores at age 6, having high blood pressure as a result of that, which remains high over the following three years. Regression to the mean is an issue in longitudinal studies, and may partly explain the findings where participants were classified and examined based on baseline measures (e.g., weight status) [45]. The study only included children from one area of the UK, and these were predominantly of white British ethnicity, so our findings may not be representative of other populations.

## Conclusions

Our findings suggest that childhood BMI may be a risk factor in the development of high blood pressure. However, the time children spend being physically active or sedentary does not appear to be strongly associated with blood pressure at this age. If our findings are replicated in other studies, including studies such as Mendelian randomisation and within sibship analyses that might be less prone to residual confounding [46], then that, together with the limited effectiveness to date of physical activity interventions on BMI [47], suggests that other interventions to prevent the development of cardiovascular disease in early life may be important.

## Supporting information

S1 Fig Frequency distributions for BMI z-score at 6 years and 9 years in overlay. (TIF)

S2 Fig Frequency distributions for MVPA minutes/day at 6 years and 9 years in overlay. (TIF)

S1 Table Prospective associations of change in BMI with blood pressure at age 9 years in the multiple imputation data (N = 685). (DOCX)

S2 Table Prospective associations of physical activity with blood pressure at age 9 years in the multiple imputation data (N = 685)*. (DOCX)

S3 Table Prospective associations of change in physical activity with blood pressure at age 9 years in the multiple imputation data (N = 685)*. (DOCX)

S4 Table Cross-sectional and prospective associations of BMI with blood pressure at age 9 years for those with complete data. (DOCX)

S5 Table Cross-sectional and prospective associations of BMI with hypertension at age 9 years for those with complete data. (DOCX)

S6 Table Prospective associations of change in BMI with blood pressure at age 9 years for those with complete data. (DOCX)

S7 Table Cross-sectional associations of physical activity with blood pressure at age 9 years for those with complete data (N = 332)*. (DOCX)

S8 Table Prospective associations of physical activity with blood pressure at age 9 years for those with complete data (N = 249)*. (DOCX)

S9 Table Prospective associations of change in physical activity with blood pressure at age 9 years for those with complete data (N *=* 224)*. (DOCX)
