# Supplementary figures and images for: A longitudinal study of the associations of children's body mass index and physical activity with blood pressure
Source: PLoS One. 2017 Dec 19;12(12):e0188618. doi: 10.1371/journal.pone.0188618 (PMC5736182; doi:10.1371/journal.pone.0188618)

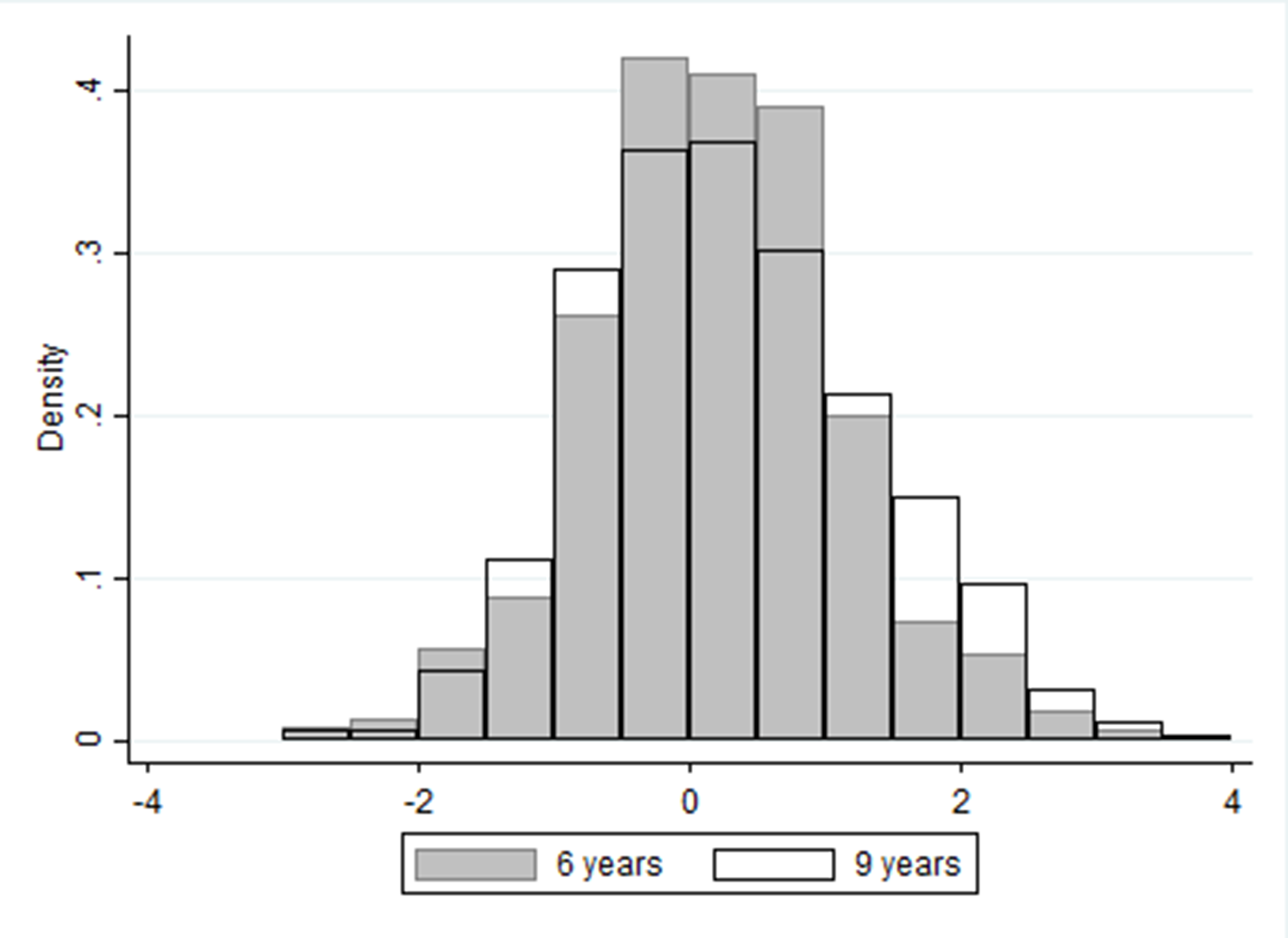

Supplement: S1 Fig — (TIF) [file pone.0188618.s001.tif]

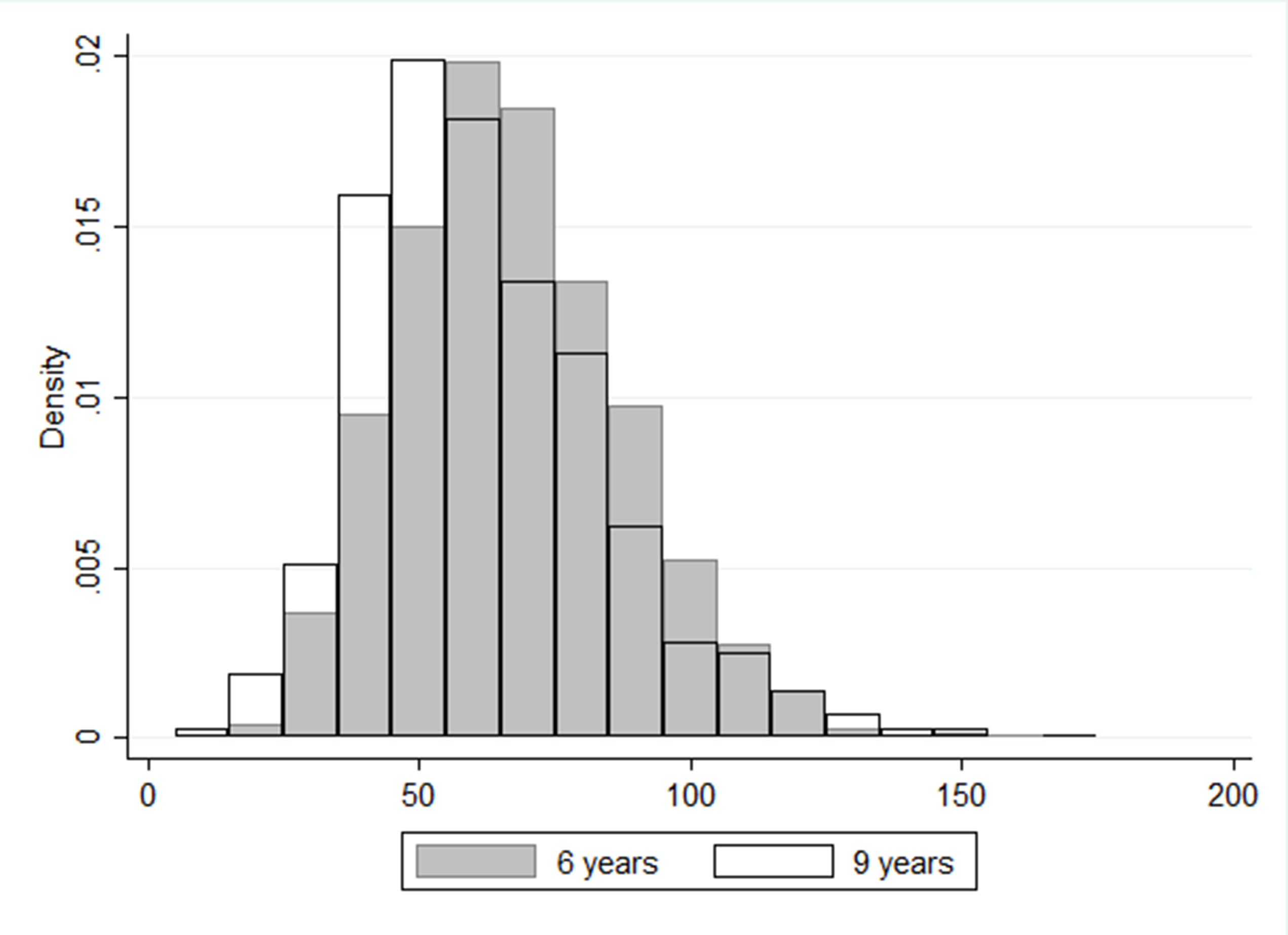

Supplement: S2 Fig — (TIF) [file pone.0188618.s002.tif]
