# Supplementary material for: A longitudinal study of the associations of children's body mass index and physical activity with blood pressure
Source: PLoS One. 2017 Dec 19;12(12):e0188618. doi: 10.1371/journal.pone.0188618 (PMC5736182; doi:10.1371/journal.pone.0188618)
Supplement: S1 Table — (DOCX) [file pone.0188618.s003.docx]

**Table S1. Prospective associations of change in BMI with blood pressure at age 9 years in the multiple imputation data (N=685)**

| **Exposure** | | **Systolic blood pressure (mmHg) at 9 years** | | | **Diastolic blood pressure (mmHg) at 9 years** | | |
| --- | --- | --- | --- | --- | --- | --- | --- |
|  |  | **All (N=685)**  Mean difference  (95% CI) | **Boys (N=323)**  Mean difference  (95% CI) | **Girls (N=362)**  Mean difference  (95% CI) | **All (N=685)**  Mean difference  (95% CI) | **Boys (N=323)**  Mean difference  (95% CI) | **Girls (N=362)**  Mean difference  (95% CI) |
| **Change in BMI z-score between 6 to 9 years (per SD of BMI)*** | | | |  |  |  |  |
|  | Model 1 | 0.40 (-0.88, 1.67) | 0.95 (-0.66, 2.57) | -0.05 (-2.06, 1.96) | 1.08 (-0.19, 2.35) | 1.03 (-0.59, 2.66) | 1.18 (-0.67, 3.04) |
|  | Model 2 | 0.68 (-0.61, 1.98) | 1.16 (-0.45, 2.78) | 0.04 (-2.11, 2.19) | 1.23 (-0.09, 2.54) | 1.05 (-0.57, 2.68) | 1.26 (-0.71, 3.24) |
| P for gender interaction | | 0.55 |  |  | 0.69 |  |  |
| **Change in BMI category between 6 to 9 years**^†^ | | | |  |  |  |  |
| Model 1 | Normal-Normal (ref) | 0 | 0 | 0 | 0 | 0 | 0 |
|  | Normal-Overweight | 3.49 (1.20, 5.79) | 7.35 (3.22, 11.49) | 0.95 (-2.27, 4.17) | 3.29 (0.91, 5.67) | 7.24 (2.97, 11.51) | 0.38 (-2.61, 3.37) |
|  | Overweight -Normal | 3.85 (-1.74, 9.43) | 3.76 (-3.19, 10.72) | 2.64 (-5.89, 11.17) | 3.53 (-2.02, 9.08) | 4.49 (-2.70, 11.67) | 1.79 (-5.46, 9.04) |
|  | Overweight -Overweight | 1.69 (-1.58, 4.97) | -1.17 (-5.64, 3.31) | 3.64 (0.13, 7.15) | 1.78 (-0.88, 4.45) | -2.45 (-6.62, 1.73) | 4.08 (1.15, 7.02) |
| Model 2 | Normal-Normal (ref) | 0 | 0 | 0 | 0 | 0 | 0 |
|  | Normal-Overweight | 3.98 (1.68, 6.28) | 7.75 ( 3.59, 11.91) | 1.01 (-2.73, 4.76) | 3.56 (1.14, 5.98) | 7.38 (3.31, 11.46) | 0.44 (-2.92, 3.79) |
|  | Overweight -Normal | 4.07 (-1.56, 9.70) | 4.78 (-2.18, 11.75) | 2.26 (-6.31, 10.83) | 3.73 (-1.88, 9.34) | 5.38 (-2.16, 12.93) | 1.45 (-5.55, 8.45) |
|  | Overweight -Overweight | 2.28 (-1.00, 5.56) | -0.09 (-4.46, 4.27) | 3.68 (0.06, 7.30) | 2.13 (-0.60, 4.87) | -1.82 (-6.10, 2.45) | 4.17 (0.95, 7.39) |
| P for gender interaction | | 0.03 |  |  | 0.004 |  |  |

* Model 1 is adjusted for BMI z-score at age 6 years; Model 2 is additionally adjusted for IMD score at 6 years, maternal and paternal BMI at 6 years and parental high blood pressure

^†^ Model 1 is unadjusted; Model 2 is additionally adjusted for IMD score at 6 years, maternal and paternal BMI at 6 years and parental high blood pressure
