# Supplementary material for: A longitudinal study of the associations of children's body mass index and physical activity with blood pressure
Source: PLoS One. 2017 Dec 19;12(12):e0188618. doi: 10.1371/journal.pone.0188618 (PMC5736182; doi:10.1371/journal.pone.0188618)
Supplement: S2 Table — (DOCX) [file pone.0188618.s004.docx]

**Table S2. Prospective associations of physical activity with blood pressure at age 9 years in the multiple imputation data (N=685)***

| **Exposure** | | **Systolic blood pressure (mmHg) at 9 years** | | | **Diastolic blood pressure (mmHg) at 9 years** | | |
| --- | --- | --- | --- | --- | --- | --- | --- |
|  |  | Mean difference | 95% confidence interval | P-value | Mean difference | 95% confidence interval | P-value |
| **Counts per minute at 6 years (per 100 cpm)** | | |  |  |  |  |  |
|  | Model 1 | -0.18 | (-0.78, 0.42) | 0.54 | 0.10 | (-0.46, 0.65) | 0.72 |
|  | Model 2 | -0.17 | (-0.78, 0.43) | 0.56 | 0.09 | (-0.48, 0.65) | 0.75 |
|  | Model 3 | -0.17 | (-0.78, 0.43) | 0.56 | 0.09 | (-0.48, 0.66) | 0.75 |
| **MVPA at 6 years (per 10 mins/day)** | | |  |  |  |  |  |
|  | Model 1 | 0.05 | (-0.44, 0.54) | 0.85 | 0.22 | (-0.28, 0.71) | 0.38 |
|  | Model 2 | 0.05 | (-0.45, 0.54) | 0.85 | 0.21 | (-0.29, 0.71) | 0.40 |
|  | Model 3 | 0.06 | (-0.45, 0.56) | 0.82 | 0.22 | (-0.30, 0.74) | 0.39 |
| **Sedentary time at 6 years (per 10 mins/day)** | | |  |  |  |  |  |
|  | Model 1 | 0.07 | (-0.09, 0.24) | 0.37 | -0.04 | (-0.19, 0.11) | 0.61 |
|  | Model 2 | 0.07 | (-0.10, 0.24) | 0.40 | -0.04 | (-0.19, 0.12) | 0.64 |
|  | Model 3 | 0.08 | (-0.10, 0.25) | 0.36 | -0.03 | (-0.19, 0.13) | 0.72 |

* Model 1 is adjusted for the child’s gender, age and height at age 6 years; Model 2 is additionally adjusted for household IMD score, maternal BMI, paternal BMI at 6 years and parental high blood pressure; Model 3 is additionally adjusted for mediation by the child’s BMI z-score at 9 years
