# Supplementary material for: A longitudinal study of the associations of children's body mass index and physical activity with blood pressure
Source: PLoS One. 2017 Dec 19;12(12):e0188618. doi: 10.1371/journal.pone.0188618 (PMC5736182; doi:10.1371/journal.pone.0188618)
Supplement: S3 Table — (DOCX) [file pone.0188618.s005.docx]

**Table S3. Prospective associations of change in physical activity with blood pressure at age 9 years in the multiple imputation data (N=685)***

| **Exposure** | | **Systolic blood pressure (mmHg) at Year 4** | | | **Diastolic blood pressure (mmHg) at Year 4** | | |
| --- | --- | --- | --- | --- | --- | --- | --- |
|  |  | Mean difference | 95% confidence interval | P-value | Mean difference | 95% confidence interval | P-value |
| **Change in counts per minute between 6 to 9 years (per 100 cpm)** | | | |  |  |  |  |
|  | Model 1 | 0.12 | (-0.38, 0.63) | 0.62 | -0.14 | (-0.58, 0.29) | 0.51 |
|  | Model 2 | 0.09 | (-0.42, 0.60) | 0.72 | -0.16 | (-0.59, 0.27) | 0.45 |
|  | Model 3 | 0.10 | (-0.42, 0.62) | 0.69 | -0.15 | (-0.58, 0.29) | 0.50 |
| **Change in MVPA between 6 to 9 years (per 10 mins/day)** | | | |  |  |  |  |
|  | Model 1 | 0.18 | (-0.32, 0.69) | 0.47 | 0.03 | (-0.44, 0.49) | 0.90 |
|  | Model 2 | 0.16 | (-0.36, 0.68) | 0.53 | 0.01 | (-0.46, 0.48) | 0.96 |
|  | Model 3 | 0.21 | (-0.32, 0.75) | 0.43 | 0.07 | (-0.41, 0.54) | 0.78 |
| **Change in sedentary time between 6 to 9 years (per 10 mins/day)** | | | |  |  |  |  |
|  | Model 1 | -0.01 | (-0.09, 0.07) | 0.80 | 0.05 | (-0.02, 0.12) | 0.19 |
|  | Model 2 | 0.00 | (-0.08, 0.07) | 0.92 | 0.05 | (-0.03, 0.12) | 0.19 |
|  | Model 3 | 0.00 | (-0.07, 0.08) | 0.99 | 0.05 | (-0.02, 0.12) | 0.16 |

* Model 1 is adjusted for the child’s gender, age, height and CPM/MVPA/sedentary time at age 6 years; Model 2 is additionally adjusted for household IMD score, maternal BMI, paternal BMI at 6 years and parental high blood pressure; Model 3 is additionally adjusted for mediation by the child’s BMI z-score at 9 years
