# Supplementary material for: A longitudinal study of the associations of children's body mass index and physical activity with blood pressure
Source: PLoS One. 2017 Dec 19;12(12):e0188618. doi: 10.1371/journal.pone.0188618 (PMC5736182; doi:10.1371/journal.pone.0188618)
Supplement: S4 Table — (DOCX) [file pone.0188618.s006.docx]

**Table S4. Cross-sectional and prospective associations of BMI with blood pressure at age 9 years for those with complete data**

| **Exposure** | | **Systolic blood pressure (mmHg) at 9 years** | | | **Diastolic blood pressure (mmHg) at 9 years** | | |
| --- | --- | --- | --- | --- | --- | --- | --- |
|  |  | Mean difference  (95% CI) | Mean difference  (95% CI) | Mean difference  (95% CI) | Mean difference  (95% CI) | Mean difference  (95% CI) | Mean difference  (95% CI) |
| **BMI z-score at 9 years (per SD of BMI)^*^** | | All (N=370) | Boys (N=183) | Girls (N=187) | All (N=370) | Boys (N=183) | Girls (N=187) |
|  | Model 1 | 0.96 (-0.32, 2.25) | 0.92 (-0.74, 2.58) | 1.07 (-0.59, 2.73) | 0.76 (-0.56, 2.08) | 0.63 (-0.90, 2.16) | 0.83 (-0.91, 2.56) |
|  | Model 2 | 0.71 (-0.74, 2.16) | 0.96 (-0.99, 2.91) | 0.71 (-1.23, 2.66) | 0.72 (-0.70, 2.13) | 0.83 (-0.92, 2.57) | 0.75 (-1.11, 2.60) |
| P value for gender interaction | | 0.85 |  |  | 0.78 |  |  |
| **Overweight (vs normal weight) at 9 years^*^** | | |  |  |  |  |  |
|  | Model 1 | 1.66 (-1.67, 5.00) | -1.27 (-6.00, 3.45) | 4.00 (0.37, 7.63) | 1.05 (-1.59, 3.70) | -2.63 (-6.36, 1.11) | 3.32 (0.03, 6.62) |
|  | Model 2 | 0.91 (-2.69, 4.51) | -2.17 (-7.62, 3.27) | 3.61 (-0.54, 7.76) | 0.72 (-2.19, 3.63) | -3.33 (-7.63, 0.97) | 3.44 (-0.17, 7.05) |
| P value for gender interaction | | 0.06 |  |  | 0.02 |  |  |
| **BMI z-score at 6 years (per SD of BMI)**^†^ | | All (N=275) | Boys (N=139) | Girls (N=136) | All (N=275) | Boys (N=139) | Girls (N=136) |
|  | Model 1 | 1.98 (0.15, 3.81) | 2.58 (-0.18, 5.34) | 1.59 (-0.66, 3.84) | 1.51 (0.06, 2.95) | 2.03 (-0.12, 4.17) | 1.05 (-0.64, 2.75) |
|  | Model 2 | 1.94 (0.09, 3.78) | 2.73 (-0.32, 5.78) | 1.52 (-0.79, 3.83) | 1.51 (0.08, 2.94) | 1.94 (-0.44, 4.32) | 1.36 (-0.36, 3.08) |
| P value for gender interaction | | 0.56 |  |  | 0.53 |  |  |
| **Overweight (vs normal weight) at 6 years**^†^ | | |  |  |  |  |  |
|  | Model 1 | 4.10 (-1.02, 9.22) | 2.80 (-5.21, 10.82) | 5.40 (0.50, 10.30) | 3.36 (-1.11, 7.83) | 2.72 (-5.33, 10.78) | 3.97 (-0.70, 8.64) |
|  | Model 2 | 4.36 (-1.23, 9.95) | 2.48 (-6.09, 11.04) | 5.75 (0.92, 10.59) | 3.40 (-1.28, 8.09) | 2.19 (-6.48, 10.86) | 4.67 (-0.12, 9.45) |
| P value for gender interaction | | 0.48 |  |  | 0.72 |  |  |

* Model 1 is unadjusted; Model 2 is adjusted for the household IMD score, maternal BMI, paternal BMI at 9 years and parental high blood pressure

^†^ Model 1 is unadjusted; Model 2 is adjusted for the household IMD score, maternal BMI, paternal BMI at 6 years and parental high blood pressure
