# Supplementary material for: A longitudinal study of the associations of children's body mass index and physical activity with blood pressure
Source: PLoS One. 2017 Dec 19;12(12):e0188618. doi: 10.1371/journal.pone.0188618 (PMC5736182; doi:10.1371/journal.pone.0188618)
Supplement: S5 Table — (DOCX) [file pone.0188618.s007.docx]

**Table S5. Cross-sectional and prospective associations of BMI with hypertension at age 9 years for those with complete data**

| **Exposure** | | **Systolic hypertension at 9 years** | | | **Diastolic hypertension at 9 years** | | |
| --- | --- | --- | --- | --- | --- | --- | --- |
|  |  | Odds ratio  (95% CI) | Odds ratio  (95% CI) | Odds ratio  (95% CI) | Odds ratio  (95% CI) | Odds ratio  (95% CI) | Odds ratio  (95% CI) |
| **BMI z-score at 9 years (per SD of BMI)^*^** | | All (N=370) | Boys (N=183) | Girls (N=187) | All (N=370) | Boys (N=183) | Girls (N=187) |
|  | Model 1 | 1.15 (0.80, 1.64) | 1.17 (0.83, 1.64) | 1.11 (0.69, 1.80) | 1.15 (0.83, 1.59) | 1.08 (0.78, 1.50) | 1.18 (0.78, 1.77) |
|  | Model 2 | 1.08 (0.71, 1.64) | 1.12 (0.72, 1.72) | 1.06 (0.62, 1.81) | 1.16 (0.80, 1.70) | 1.06 (0.67, 1.69) | 1.20 (0.78, 1.86) |
| P value for gender interaction | | 0.88 |  |  | 0.67 |  |  |
| **Overweight (vs normal weight) at 9 years^*^** | | |  |  |  |  |  |
|  | Model 1 | 1.45 (0.68, 3.08) | 0.60 (0.13, 2.72) | 2.03 (0.84, 4.95) | 1.46 (0.76, 2.78) | 0.60 (0.17, 2.08) | 2.02 (0.98, 4.15) |
|  | Model 2 | 1.28 (0.53, 3.09) | 0.42 (0.08, 2.33) | 2.11 (0.75, 5.91) | 1.49 (0.69, 3.22) | 0.49 (0.12, 2.10) | 2.32 (1.04, 5.14) |
| P value for gender interaction | | 0.17 |  |  | 0.08 |  |  |
| **BMI z-score at 6 years (per SD of BMI)**^†^ | | All (N=275) | Boys (N=139) | Girls (N=136) | All (N=275) | Boys (N=139) | Girls (N=136) |
|  | Model 1 | 1.45 (0.93, 2.26) | 1.67 (1.02, 2.75) | 1.24 (0.61, 2.55) | 1.57 (1.12, 2.18) | 2.20 (1.32, 3.67) | 1.14 (0.70, 1.87) |
|  | Model 2 | 1.46 (0.92, 2.30) | 1.62 (0.91, 2.88) | 1.16 (0.52, 2.60) | 1.62 (1.16, 2.25) | 2.21 (1.18, 4.16) | 1.17 (0.69, 1.98) |
| P value for gender interaction | | 0.53 |  |  | 0.09 |  |  |
| **Overweight (vs normal weight) at 6 years^**^** | | |  |  |  |  |  |
|  | Model 1 | 2.26 (0.93, 5.49) | 1.03 (0.20, 5.22) | 3.78 (1.50, 9.56) | 2.27 (1.18, 4.37) | 2.41 (0.71, 8.18) | 2.11 (0.91, 4.93) |
|  | Model 2 | 2.33 (0.92, 5.95) | 0.87 (0.16, 4.70) | 4.71 (1.85, 12.02) | 2.38 (1.15, 4.92) | 2.18 (0.60, 7.94) | 2.50 (1.03, 6.04) |
| P value for gender interaction | | 0.13 |  |  | 0.96 |  |  |

* Model 1 is unadjusted; Model 2 is adjusted for the household IMD score, maternal BMI, paternal BMI at 9 years and parental high blood pressure

^†^ Model 1 is unadjusted; Model 2 is adjusted for the household IMD score, maternal BMI, paternal BMI at 6 years and parental high blood pressure
