# Supplementary material for: A longitudinal study of the associations of children's body mass index and physical activity with blood pressure
Source: PLoS One. 2017 Dec 19;12(12):e0188618. doi: 10.1371/journal.pone.0188618 (PMC5736182; doi:10.1371/journal.pone.0188618)
Supplement: S6 Table — (DOCX) [file pone.0188618.s008.docx]

**Table S6. Prospective associations of change in BMI with blood pressure at age 9 years for those with complete data**

| **Exposure** | | **Systolic blood pressure (mmHg) at 9 years** | | | **Diastolic blood pressure (mmHg) at 9 years** | | |
| --- | --- | --- | --- | --- | --- | --- | --- |
|  |  | **All (N=275)**  Mean difference  (95% CI) | **Boys (N=139)**  Mean difference  (95% CI) | **Girls (N=136)**  Mean difference  (95% CI) | **All (N=275)**  Mean difference  (95% CI) | **Boys (N=139)**  Mean difference  (95% CI) | **Girls (N=136)**  Mean difference  (95% CI) |
| **Change in BMI z-score between 6 to 9 years (per SD of BMI)^*^** | | | |  |  |  |  |
|  | Model 1 | 0.01 (-2.04, 2.06) | -0.49 (-3.20, 2.22) | 0.86 (-2.11, 3.82) | 0.33 (-1.48, 2.14) | 0.14 (-1.96, 2.23) | 0.80 (-2.43, 4.02) |
|  | Model 2 | 0.05 (-2.08, 2.18) | -0.60 (-3.68, 2.48) | 1.42 (-2.06, 4.90) | 0.43 (-1.40, 2.26) | -0.08 (-2.21, 2.05) | 1.29 (-2.28, 4.86) |
| P for gender interaction | | 0.46 |  |  | 0.71 |  |  |
| **Change in BMI category between 6 to 9 years**^†^ | | | |  |  |  |  |
| Model 1 | Normal-Normal (ref) | 0 | 0 | 0 | 0 | 0 | 0 |
|  | Normal-Overweight | 2.48 (-0.73, 5.68) | 4.70 (-0.81, 10.20) | 1.45 (-3.08, 5.98) | 1.15 (-1.47, 3.76) | 0.79 (-3.36, 4.94) | 1.60 (-2.20, 5.41) |
|  | Overweight -Normal | 10.34 (-2.32, 23.00) | 12.36 (-1.03, 25.75) | -5.85 (-7.41, -4.30) | 12.72 (-0.26, 25.71) | 13.95 (-0.98, 28.87) | 3.15 (1.64, 4.67) |
|  | Overweight -Overweight | 3.05 (-2.60, 8.71) | -1.76 (-9.26, 5.74) | 6.19 (0.39, 11.99) | 1.41 (-2.77, 5.59) | -3.29 (-9.63, 3.06) | 4.26 (-0.74, 9.26) |
| Model 2 | Normal-Normal (ref) | 0 | 0 | 0 | 0 | 0 | 0 |
|  | Normal-Overweight | 2.26 (-1.12, 5.64) | 4.38 (-2.33, 11.09) | 2.32 (-3.24, 7.89) | 0.97 (-2.11, 4.05) | -0.22 (-4.99, 4.56) | 2.94 (-1.36, 7.25) |
|  | Overweight -Normal | 10.49 (-2.47, 23.45) | 12.67 (-1.00, 26.34) | -6.80 (-8.40, -5.21) | 12.65 (-0.36, 25.66) | 13.79 (-1.47, 29.05) | 2.41 (0.81, 4.02) |
|  | Overweight -Overweight | 3.02 (-2.93, 8.98) | -2.43 (-10.91, 6.06) | 6.89 (0.86, 12.92) | 1.36 (-3.06, 5.79) | -4.58 (-11.64, 2.48) | 5.33 (-0.02, 10.68) |
| P for gender interaction | | 0.009 |  |  | 0.11 |  |  |

* Model 1 is adjusted for BMI z-score at age 6 years; Model 2 is additionally adjusted for IMD score at 6 years, maternal and paternal BMI at 6 years and parental high blood pressure

^†^ Model 1 is unadjusted; Model 2 is additionally adjusted for IMD score at age 6 years, maternal and paternal BMI at 6 years and parental high blood pressure
