# Supplementary material for: A longitudinal study of the associations of children's body mass index and physical activity with blood pressure
Source: PLoS One. 2017 Dec 19;12(12):e0188618. doi: 10.1371/journal.pone.0188618 (PMC5736182; doi:10.1371/journal.pone.0188618)
Supplement: S7 Table — (DOCX) [file pone.0188618.s009.docx]

**Table S7. Cross-sectional associations of physical activity with blood pressure at age 9 years for those with complete data (N=332)***

| **Exposure** | | **Systolic blood pressure (mmHg) at 9 years** | | | **Diastolic blood pressure (mmHg) at 9 years** | | |
| --- | --- | --- | --- | --- | --- | --- | --- |
|  |  | Mean difference | 95% confidence interval | P-value | Mean difference | 95% confidence interval | P-value |
| **Counts per minute at 9 years (per 100 cpm)** | | |  |  |  |  |  |
|  | Model 1 | -0.17 | (-0.82, 0.48) | 0.60 | -0.39 | (-0.97, 0.18) | 0.18 |
|  | Model 2 | -0.15 | (-0.79, 0.49) | 0.64 | -0.40 | (-0.97, 0.18) | 0.17 |
|  | Model 3 | -0.13 | (-0.79, 0.52) | 0.69 | -0.37 | (-0.97, 0.23) | 0.22 |
| **MVPA at 9 years (per 10 mins/day)** | |  |  |  |  |  |  |
|  | Model 1 | -0.02 | (-0.71, 0.67) | 0.95 | -0.18 | (-0.77, 0.41) | 0.54 |
|  | Model 2 | 0.00 | (-0.69, 0.69) | 0.997 | -0.18 | (-0.76, 0.40) | 0.54 |
|  | Model 3 | 0.03 | (-0.70, 0.75) | 0.94 | -0.13 | (-0.75, 0.48) | 0.66 |
| **Sedentary time at 9 years (per 10 mins/day)** | | |  |  |  |  |  |
|  | Model 1 | 0.08 | (-0.03, 0.19) | 0.14 | 0.10 | (0.00, 0.19) | 0.05 |
|  | Model 2 | 0.08 | (-0.02, 0.19) | 0.13 | 0.10 | (0.01, 0.19) | 0.03 |
|  | Model 3 | 0.08 | (-0.02, 0.19) | 0.12 | 0.10 | (0.01, 0.19) | 0.02 |

* Model 1 is adjusted for the child’s gender, age and height at age 9 years; Model 2 is additionally adjusted for household IMD score, maternal BMI, paternal BMI at age 9 years and parental high blood pressure; Model 3 is additionally adjusted for mediation by the child’s BMI z-score at 9 years
