# Supplementary material for: A longitudinal study of the associations of children's body mass index and physical activity with blood pressure
Source: PLoS One. 2017 Dec 19;12(12):e0188618. doi: 10.1371/journal.pone.0188618 (PMC5736182; doi:10.1371/journal.pone.0188618)
Supplement: S8 Table — (DOCX) [file pone.0188618.s010.docx]

**Table S8. Prospective associations of physical activity with blood pressure at age 9 years for those with complete data (N=249)***

| **Exposure** | | **Systolic blood pressure (mmHg) at 9 years** | | | **Diastolic blood pressure (mmHg) at 9 years** | | |
| --- | --- | --- | --- | --- | --- | --- | --- |
|  |  | Mean difference | 95% confidence interval | P-value | Mean difference | 95% confidence interval | P-value |
| **Counts per minute at 6 years (per 100 cpm)** | | |  |  |  |  |  |
|  | Model 1 | -0.34 | (-1.16, 0.47) | 0.40 | 0.04 | (-0.66, 0.74) | 0.91 |
|  | Model 2 | -0.41 | (-1.20, 0.39) | 0.31 | -0.01 | (-0.70, 0.68) | 0.98 |
|  | Model 3 | -0.48 | (-1.29, 0.33) | 0.24 | -0.07 | (-0.75, 0.61) | 0.84 |
| **MVPA at 6 years (per 10 mins/day)** | |  |  |  |  |  |  |
|  | Model 1 | -0.03 | (-0.81, 0.75) | 0.94 | 0.18 | (-0.55, 0.90) | 0.63 |
|  | Model 2 | -0.07 | (-0.84, 0.69) | 0.85 | 0.15 | (-0.57, 0.86) | 0.68 |
|  | Model 3 | -0.12 | (-0.91, 0.66) | 0.75 | 0.10 | (-0.62, 0.81) | 0.78 |
| **Sedentary time at 6 years (per 10 mins/day)** | | |  |  |  |  |  |
|  | Model 1 | 0.03 | (-0.28, 0.33) | 0.86 | -0.08 | (-0.33, 0.17) | 0.50 |
|  | Model 2 | 0.05 | (-0.26, 0.35) | 0.75 | -0.06 | (-0.31, 0.18) | 0.60 |
|  | Model 3 | 0.06 | (-0.24, 0.36) | 0.70 | -0.06 | (-0.30, 0.18) | 0.64 |

* Model 1 is adjusted for the child’s gender, age and height at age 6 years; Model 2 is additionally adjusted for household IMD score, maternal BMI, paternal BMI at age 6 years and parental high blood pressure; Model 3 is additionally adjusted for mediation by the child’s BMI z-score at 9 years
