# Supplementary material for: A longitudinal study of the associations of children's body mass index and physical activity with blood pressure
Source: PLoS One. 2017 Dec 19;12(12):e0188618. doi: 10.1371/journal.pone.0188618 (PMC5736182; doi:10.1371/journal.pone.0188618)
Supplement: S9 Table — (DOCX) [file pone.0188618.s011.docx]

**Table S9. Prospective associations of change in physical activity with blood pressure at age 9 years for those with complete data (N=224)***

| **Exposure** | | | | **Systolic blood pressure (mmHg) at 9 years** | | | **Diastolic blood pressure (mmHg) at 9 years** | | |
| --- | --- | --- | --- | --- | --- | --- | --- | --- | --- |
|  |  |  |  | Mean difference | 95% confidence interval | P-value | Mean difference | 95% confidence interval | P-value |
| **Change in counts per minute between 6 to 9 years (per 100 cpm)** | | |  | | |  |  |  |  |
|  | Model 1 | | | -0.75 | (-1.57, 0.07) | 0.07 | -1.03 | (-1.74, -0.32) | 0.006 |
|  | Model 2 | | | -0.72 | (-1.51, 0.08) | 0.08 | -1.04 | (-1.76, -0.32) | 0.005 |
|  | Model 3 | | | -0.65 | (-1.49, 0.19) | 0.13 | -0.99 | (-1.75, -0.23) | 0.01 |
| **Change in MVPA between 6 to 9 years (per 10 mins/day)** | |  | | |  |  |  |  |  |
|  | Model 1 | | | -0.57 | (-1.53, 0.39) | 0.24 | -0.74 | (-1.47, -0.01) | 0.05 |
|  | Model 2 | | | -0.51 | (-1.47, 0.46) | 0.30 | -0.72 | (-1.47, 0.02) | 0.06 |
|  | Model 3 | | | -0.43 | (-1.43, 0.58) | 0.40 | -0.66 | (-1.45, 0.12) | 0.10 |
| **Change in sedentary time between 6 to 9 years (per 10 mins/day)** | | |  | | |  |  |  |  |
|  | Model 1 | | | 0.11 | (-0.02, 0.24) | 0.09 | 0.14 | (0.03, 0.26) | 0.02 |
|  | Model 2 | | | 0.12 | (0.00, 0.24) | 0.05 | 0.15 | (0.04, 0.26) | 0.007 |
|  | Model 3 | | | 0.11 | (-0.00, 0.23) | 0.06 | 0.15 | (0.04, 0.25) | 0.008 |

* Model 1 is adjusted for the child’s gender, age, height and CPM/MVPA/sedentary time at age 6 years; Model 2 is additionally adjusted for household IMD score, maternal BMI, paternal BMI at age 6 years and parental high blood pressure; Model 3 is additionally adjusted for mediation by the child’s BMI z-score at 9 years
